# Diversity in olfactory receptor repertoires is associated with dietary specialization in a genus of frugivorous bat

**DOI:** 10.1093/g3journal/jkab260

**Published:** 2021-07-22

**Authors:** Laurel R Yohe, Leith B Leiser-Miller, Zofia A Kaliszewska, Paul Donat, Sharlene E Santana, Liliana M Dávalos

**Affiliations:** 1 Department of Earth and Planetary Sciences, Yale University, New Haven, CT 06511, USA; 2 Department of Ecology and Evolution, Stony Brook University, Stony Brook, NY 11794, USA; 3 Department of Biology, University of Washington, Seattle, WA 98195, USA; 4 Burke Museum of Natural History and Culture, University of Washington, Seattle, WA 98105, USA; 5 Consortium for Inter-Disciplinary Environmental Research, School of Marine and Atmospheric Sciences, Stony Brook University, Stony Brook, NY 11794, USA

**Keywords:** olfactory receptor, *Piper*, *Carollia*, Phyllostomidae, bats, gene duplication

## Abstract

Mammalian *olfactory receptor* genes (*OR*s) are a diverse family of genes encoding proteins that directly interact with environmental chemical cues. *OR*s evolve via gene duplication in a birth-death fashion, neofunctionalizing and pseudogenizing over time. Olfaction is a primary sense used for food detection in plant-visiting bats, but the relationship between dietary specialization and *OR* repertoire diversity is unclear. Within neotropical Leaf-nosed bats (Phyllostomidae), many lineages are plant specialists, and some have a distinct *OR* repertoire compared to insectivorous species. Yet, whether specialization on particular plant genera is associated with the evolution of specialized, less diverse *OR* repertoires has never been tested. Using targeted sequence capture, we sequenced the *OR* repertoires of three sympatric species of short-tailed fruit bats (*Carollia*), which vary in their degree of specialization on the fruits of *Piper* plants. We characterized orthologous *vs* duplicated receptors among *Carollia* species, and explored the diversity and redundancy of the receptor gene repertoire. At the species level, the most dedicated *Piper* specialist, *Carollia castanea*, had lower *OR* diversity compared to the two generalists (*C. sowelli* and *C. perspicillata*), but we discovered a few unique sets of *OR*s within *C. castanea* with high redundancy of similar gene duplicates. These unique receptors potentially enable *C. castanea* to detect *Piper* fruit odorants better than its two congeners. *Carollia perspicillata*, the species with the most generalist diet, had a higher diversity of intact receptors, suggesting the ability to detect a wider range of odorant molecules. Variation among *OR*s may be a factor in the coexistence of these sympatric species, facilitating the exploitation of different plant resources. Our study sheds light on how gene duplication and changes in *OR* diversity may play a role in dietary adaptations and underlie ecological interactions between bats and plants.

## Introduction

The fitness of an animal is dependent on finding food, locating mates, and avoiding predation. Because of their relevance to fitness and the ubiquity of chemosensation in animals, biochemical and cellular mechanisms underlying the sense of smell are excellent targets for natural selection (Hayden *et al.* 2010; Niimura 2012; Nikaido *et al.* 2013). To perceive a scent, odorant molecules within a chemical bouquet bind to olfactory receptor (*OR*) proteins in a combinatorial fashion (Malnic *et al.* 1999; Nara *et al.* 2011; Kurian *et al.* 2020), precipitating a signaling cascade that ultimately transmits the odorant information to the brain. The complexity of chemical odorant bouquets coupled with both the promiscuity of the ligand-receptor relationship and the combinatorial neural encoding of olfactory cues contribute to the immense challenge of identifying ligands and their receptors, and few receptors have been “de-orphaned” outside of model organisms. Nonetheless, each individual olfactory neuron expresses a unique *OR* allele; thus, the larger the intact *OR* repertoire, the larger the combination of different odorants an organism can sense (Rodriguez 2013). This direct interaction with environmental signals suggests natural selection likely fine-tunes *OR* binding motifs to optimally detect chemical cues relevant to fitness. However, deciphering the connection between *OR*s and the ecology of animals has proved challenging because *OR*s evolve through paralogous duplication and the chemical cues necessary to elicit olfactory responses are complex (Yohe and Brand 2018).

*OR*s, as well as many other chemosensory receptor genes, evolve in a birth-death manner, such that genes are constantly duplicating and pseudogenizing through time (Nei and Rooney 2005). This genetic mechanism of change has led to extraordinary diversity amongst chemoreceptor genes, making them among the largest and fastest-evolving protein-coding gene families in the vertebrate genome (Niimura and Nei 2007; Nei *et al.* 2008; Niimura 2013; Yohe *et al.* 2020b). Mammalian *OR* genes, in particular, are ∼900bp-long, intronless genes that encode seven-transmembrane G-protein coupled receptors (Dulac and Axel 1995). In mammals, counts of intact *OR* gene copies and *OR* pseudogenes can vary by orders of magnitude (Niimura *et al.* 2014), from hundreds to thousands. The fate of a gene duplicate includes several potential paths (Hahn 2009; Teufel *et al.* 2016; Yohe *et al.* 2019b). First, the duplicated gene may be completely redundant and not be expressed, and thus it could accumulate a deleterious mutation that may render it a pseudogene (Eyun 2019). Second, one of the two copies may be released from purifying selection and accumulate new mutations that enable new function (Pegueroles *et al.* 2013). Third, the second copy may have a dosage effect, such that there is now increased expression of the ancestral single copy (Loehlin and Carroll 2016) and fixation of the same copy of the gene may be advantageous to fitness.

Measuring adaptation at the species level in large gene families has proven difficult because of the challenges of simultaneously tracking both orthology *vs* paralogy and the rate of adaptive substitution (Hahn 2009; Han *et al.* 2009; Yohe *et al.* 2019b). Here, we present a novel approach to understanding the evolutionary history of *OR* gene duplicates among recently diverged species. Using unrooted codon model gene trees, we first detect orthologous genes and associated paralogs and then measure diversity by applying metrics from community ecology. Ecological diversity statistics have previously been used to summarize nucleotide diversity at sites in an alignment (Lowry and Atchley 2000) or transcriptome complexity (Holding *et al.* 2021). We propose these metrics are also useful to characterize the diversity within orthologous clusters of genes and recent paralogs, and apply this method to investigate *OR* diversity and evolution in three sympatric species of short-tailed fruit bats (*Carollia* spp.).

**Figure 1:**
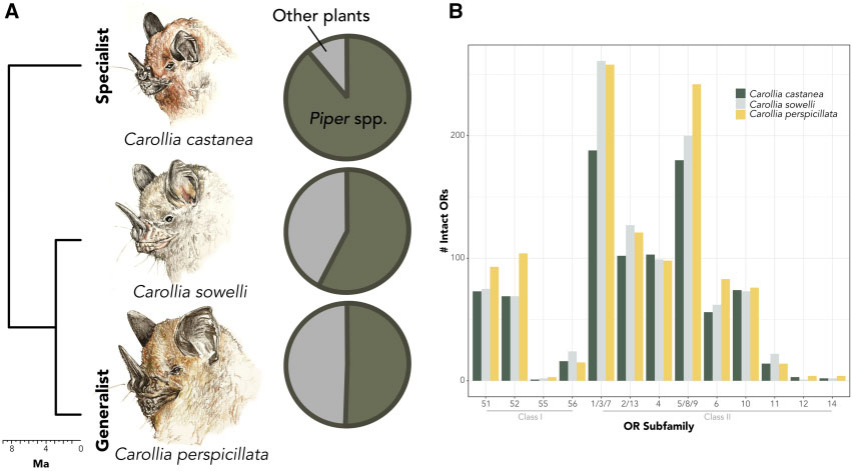
Target species of study that demonstrate varying degrees of *Piper* reliance. (A) Proportion of *Piper* species found in diet of each *Carollia* species [based on Fleming (1991); Lopez and Vaughan (2007); and Maynard *et al.* (2019)]. Estimates of 91–98% of the diet of *C. castanea* is *Piper*, while about 80% for *C. sowelli* and ∼50–80% for *C. perspicillata*. (B) Number of intact *olfactory receptor* (*OR*s) genes from sequence capture analysis within each subfamily. Illustrations by Christina M. Mauro.

*Carollia* is a genus of neotropical Leaf-nosed bats (Phyllostomidae) that diverged around 12 Ma and is composed of eight described species found throughout the Neotropics (Shi and Rabosky 2015; Rojas *et al.* 2016). The *Carollia* system is ideal for investigating a connection between ecological specialization and *OR* diversity for two reasons. First, several *Carollia* species can co-occur while showing divergent diets. The three nonsister sympatric species in our analysis consume fruits of the genus *Piper*, but the degree of *Piper* specialization varies from *Carollia castanea* feeding almost exclusively on *Piper* fruits throughout the year, to the diet of *C. perspicillata* consisting of about 50% *Piper* fruits plus a variety of other plant genera from several families, nectar from flowers, and occasionally insects; the diet of *C. sowelli* falls between that of the other two species (Figure 1A; Fleming 1991; Lopez and Vaughan 2007; Maynard *et al.* 2019). Second, behavioral assays have revealed that *Carollia* primarily use their sense of smell to locate fruiting patches and individual fruits, with echolocation used at closer range to pinpoint the target fruit before grabbing it (Thies *et al.* 1998). *Carollia* also only seem to perform feeding attempts in the presence of scent cues from *Piper* fruit (Thies *et al.* 1998; Leiser-Miller *et al.* 2020). *Piper* scent cues are remarkably diverse with strong signatures of phylogenetic overdispersion, but some chemical compounds remain conserved even in paleotropical *Piper* (Salehi *et al.* 2019; Santana *et al.* 2021) and several chemical compounds are associated with the primary diets of particular *Carollia* species (Santana *et al.* 2021). Thus, the reliance of *Carollia* on olfaction to locate *Piper* fruits (and reciprocal reliance of *Piper* on chemical cues to attract *Carollia* for seed dispersal) makes it likely that evolution has optimized the *OR* repertoires of each of these bat species for food detection. Because *C. castanea* primarily needs to locate ripe *Piper* fruits, we predict the bouquet of potential odorant ligands and therefore the diversity of respective receptors might be narrower than those of *C. perspicillata*, which need to detect not just ligands from *Piper*, but also from the diversity of other plant foods it consumes. We apply our novel approach of using ecological metrics of diversity to measure diversity among orthologous and paralogous genes to investigate how evolution has shaped *OR* repertoires in the context of specialist and generalist diets.

## Methods

### Sampling and sequencing

To test whether specialist and generalist species had distinct receptor profiles, we sequenced the *OR*s of three *Carollia* species using targeted sequence capture of probes designed from transcriptomic data. Samples were collected at La Selva Biological Station in Costa Rica during an August 2017 expedition. One male individual of each of the three *Carollia* species found at La Selva was captured on the evening of August 4, 2017 at the same locality within the station (Supplementary Table S1). Bats were trapped in mist nets and immediately placed in cloth bags prior to processing. Bats were euthanized using isoflurane and liver dissections were performed according to published video protocols (Yohe *et al.* 2019a). Bats and samples were processed in accordance with Stony Brook University Institutional Animal Care and Use Committee protocol #448712-3. Samples were collected with Costa Rica research permit CONAGEBIO #R-041-2017, exported from Costa Rica in alliance with country guidelines, and imported following U.S. Center for Disease Control and U.S. Fish & Wildlife guidelines (USFW 3-177 2018NY2190224). For the targeted bait capture, probes were designed from a previously published analysis (Yohe *et al.* 2020a). Briefly, chemosensory receptors were identified in the transcriptomes of the main olfactory epithelium in 12 species of bats and probes were subsequently designed from the diversity of these receptor transcripts. While targeted bait capture provided optimal *de novo* sequencing of *OR*s (Yohe *et al.* 2020a), it is still known to be incomplete, and interpretation of the results should consider these confounding factors. DNA was extracted from flash-frozen liver tissue stored in RNA-later using the Qiagen QIAamp DNA Micro kit (Qiagen 56304). DNA quality was assessed using 260/280 ratios in a nanodrop, and DNA was quantified using a Qubit. DNA extractions were sent to Arbor Biosciences (Ann Arbor, MI, USA) where the chemoreceptor probes were enriched for *OR*s. Amplified targets were sequenced using Illumina HiSeq sequencing technology with 100-bp paired-end reads by Arbor Biosciences (Ann Arbor, MI, USA).

### Quality control and assembly

All sequence bait capture assemblies were performed using previously published methods optimized for large multigene families (Yohe *et al.* 2020a). Briefly, raw paired-end reads were trimmed using the bbduk.sh script in the BBTools genomic tools suite, in which regions with a quality score of less than 10 were trimmed. Using the bait designs as guides for assembling the raw reads, we implemented the reads_first.py in the HybPiper toolkit (Johnson *et al.* 2016). Each lane was assembled individually, then resulting receptors were pooled, and duplicates were removed.

### Olfactory receptor annotation

In both the transcriptome assembly output and cleaned targeted bait capture output, contigs were run through the Olfactory Receptor Assigner v. 1.9.1, in which *OR*s were binned into respective subfamilies (Hayden *et al.* 2010). Pseudogenes were determined as either open reading frames disrupted by a frameshift or premature stop codon mutation or sequences less than 650 bp that would prevent a complete seven-transmembrane domain from being translated. Exact duplicates and pseudogenes were removed from the analysis.

### Alignment and gene tree inference

Each subfamily of intact receptors was aligned using the transAlign (Bininda-Emonds 2005) option in Geneious v. 10.2.3 (Kearse *et al.* 2012) with MAFFT v. 7.388 (Katoh and Standley 2013) and the FFT-NS-2 algorithm for the protein alignment. The human adenosine A2b receptor, an ancestral G-protein-coupled receptor gene, was included in each alignment in order to root the gene trees (NM_000676.2), as suggested from previous publications on mammalian *OR*s (Niimura 2013). For model selection and tree inference, stop codons were removed. Model selection was performed on each alignment using ModelOMatic v. 1.01 (Whelan *et al.* 2015), in which 75 amino acid, codon, and nucleotide evolutionary models were tested. Maximum likelihood tree inference was performed on each alignment with the estimated best-fit model using IQ-TREE v. 1.6.11 (Nguyen *et al.* 2015) with 1000 ultrafast bootstrap replicates.

### Orthogroup characterization

**Figure 2:**
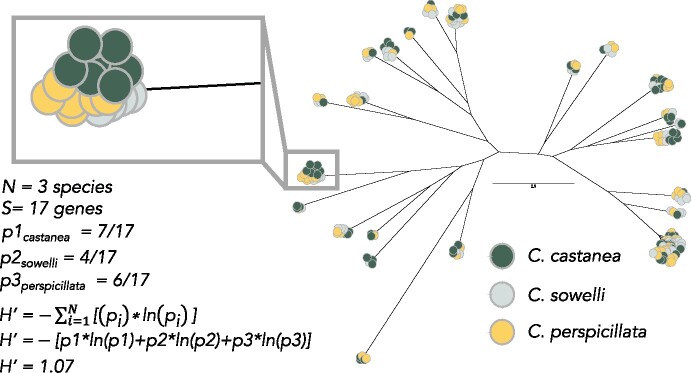
Example of gene trees and orthogroups. Inferred codon-model gene tree for *olfactory receptor* subfamily 10 (*OR*10). Each colored circle represents an *OR* gene colored by species. Larger clusters of genes are orthogroups or clusters of orthogroups that include orthologous genes and paralogs. The window inset indicates an example of an inferred orthogroup and the calculated *H’* for a single orthogroup.

To characterize orthologous *OR* genes, as well as associated duplicates accumulated both prior to (out-paralogs) and after species divergence (in-paralogs), we used an unrooted phylogenetic assessment of the gene trees for each subfamily (Ballesteros and Hormiga 2016). For each gene tree, we used the UPhO.py script within UPhO implemented with Python v. 2.7.15 with the -iP flag to track in-paralogs and minimum number of species in an orthogroup set to 1 (Ballesteros and Hormiga 2016). See Figure 2 for an example of an inferred orthogroup.

### Receptor diversity metrics

To quantify *OR* gene “diversity,” we used diversity indices commonly used in community ecology. The diversity of community composition is often assessed with species abundances (number of individuals per species) at different sites within a community. These metrics were then used to calculate community diversity. Applying this framework, we considered each *OR* subfamily as a “community” and each gene orthogroup a “site” within the community. Instead of measuring abundance as number of individuals per species within a site, we measured number of genes (duplicates) per species within the orthogroup. We can then calculated Shannon’s *H*’, or the Shannon Entropy, for total *OR* gene repertoires, as well as for each *OR* gene subfamily,

$$H’=-\sum_{i=1}^{N}\left[\left(p_{i}\right)*\text{ln}\left(p_{i}\right)\right],$$

where p is the proportion of genes in an orthogroup for species i and N is the total number of species. Figure 2 provides an example calculation for an orthogroup. Diversity indices were calculated using the diversityresult() function within the BiodiversityR v. 2.12.1 (Kindt 2016) in R. v. 4.0.2 (R Core Team 2020) for each *OR* subfamily. These values were then presented as means of each *H’* for each species or for subfamilies per species. Values of *H’* can be interpreted as an axis of diversity, such that low values of *H*’ suggest more species-level diversity and high-values of *H*’ suggest more diversity at the genus-level (among *Carollia* species). All values of *H’* are presented in natural log scale.

To statistically compare diversity values among species, we performed a phylogenetically corrected linear mixed effects model using the MCMCglmm v. 2.29 (Hadfield 2010), in which both species and *OR* subfamily were group-specific effects and the phylogenetic distance among species was measured from an inverted relatedness matrix estimated from a previously published phylogeny (Rojas *et al.* 2016). This approach allows direct comparisons of the marginal posterior distributions of parameter estimates. When 50% intervals around the median are nonoverlapping, notable differences among group coefficients were observed. To determine a threshold in which exceptional redundancy within an orthogroup exists, we performed a Poisson regression in a Bayesian framework, with the number of *OR* genes per orthogroups as the response, bat species as the covariate, and *OR* subfamily as a random effect. The MCMCglmm approach is ideal, as it accounts for exceptional residual variance that may confound our models through a built-in additive over-dispersion model. Residual variance that fails to be accounted for in the Poisson model may be derived from issues like incomplete sequencing or gene tree inference error (Hadfield 2019). The threshold of redundancy was determined through posterior predictive simulation using estimated model parameters and taking the upper limit of the 95% credible interval of the marginal distribution of predicted orthogroup abundance. All Bayesian models were run with 5 million iterations thinning every 500 samples and removing the first 1000 as burn-in.

## Results

### Olfactory receptor distribution

For each *Carollia* species, the number of intact *OR* genes were as follows: *C. castanea* 881, *C. sowelli* 1017, and *C. perspicillata* 1115 (Figure 1B; Supplementary Table S2). Figure 1B shows the abundance of *OR*s within each subfamily for each species. *OR*1/3/7 and *OR*5/8/9 showed twice the abundance relative to other subfamilies for all species, while subfamily *OR*55, *OR*12, and *OR*14 were represented by fewer paralogs relative to other subfamilies.

### Alignment and orthogroup inference

**Figure 3:**
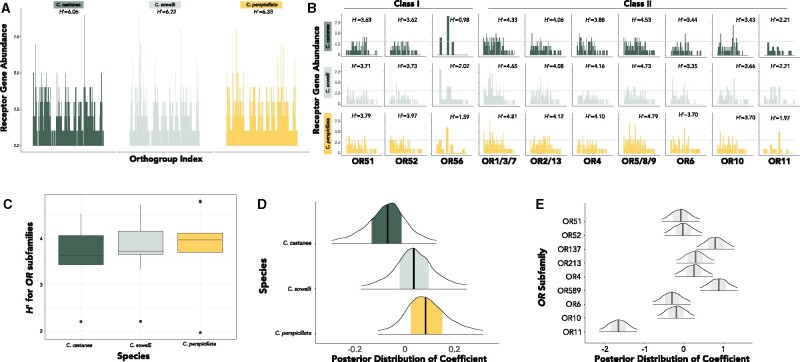
(A) Abundance profiles for each species (*C. castanea*: evergreen; *C. sowelli*: mint green; *C. perspicillata*: gold). Each bar denotes a unique orthogroup and the same orthogroup index is consistent across species for comparison; the Shannon Diversity Index (*H*’) presented for each species is pooled across all genes, not individual subfamilies. All Shannon *H’* reports are in natural log scale. (B) Abundance profiles for each species for each *OR* gene subfamily. Each bar denotes a unique orthogroup and the same orthogroup index is consistent across species for comparison. The Shannon Diversity Index (*H*’) is presented for each “community” of genes. *OR*55, *OR*12, and *OR*14, which had only a few genes in each species, are not shown. The dashed line is the estimated threshold for orthogroups in which exceptional diversity was observed. (C) Distribution of Shannon Diversity Indices (*H*’) for each *olfactory receptor* (*OR*) gene subfamily for each species. (D) Posterior distribution of diversity for each species after correcting for phylogeny and subfamily variance. (E) Posterior distribution of diversity for each *OR* subfamily after correcting for phylogeny and species variance. For panels (D,E), central black lines represent the median of the posterior, shaded regions indicated 50% of the credible interval, and 90% of the interval is shown here for clarity.

Alignments for each subfamily resulted in lengths ranging from 1065 to 1242 bp. For every alignment, codon models were the best-fit models of evolution, though the base frequencies varied (Supplementary Table S3). For all identified gene trees (*e.g.*, Figure 2), a total of 1019 orthogroups were identified (Figure 3A). The number of orthogroups per subfamily are listed in Supplementary Table S2. Alignments, gene trees, and orthogroup cluster lists are available in FigShare. Figure 3B indicates the abundance of receptors for each orthogroup for each *OR* subfamily, demonstrating how some orthogroups have higher abundances in some species *vs* others. Poisson model results found the upper limit of the posterior simulations to have a mean of 3.24 (±0.43), and thus orthogroups with 4 or more genes represented by the same species were considered outliers (Figure 3B).

### Diversity metrics

*C. perspicillata* had the most diverse *OR* repertoire among the three species (Figure 3C; *H*’ = 6.33) and *C. castanea* had the least diverse *OR* repertoire (*H’* = 6.06), while *C. sowelli* had a diversity that fell in between the other two (*H*’ = 6.22; Figure 3C). The values of *H’* represent the pooled values for the entire *OR* repertoire (not just within *OR* subfamily). After controlling for phylogeny and subfamily, *C. castanea* had notably lower diversity than *C. perspicillata* (Figure 3D). Subfamilies *OR*1/3/7 and *OR*5/8/9 had exceptionally higher diversity while *OR*11 showed notably low diversity (Figure 3E). Discernable differences in diversity can be observed in Figure 3E. Among *OR* subfamilies (Figure 3, B and D), *C. perspicillata* also consistently had the most diverse and *C. castanea* the least diverse *OR* repertoires, apart from *OR*56 (for which *C. sowelli* was most diverse) and *OR*11 (for which *C. perspicillata* was the least).

## Discussion

Ecological specialization is expected to be linked to trait diversity, with generalist species exhibiting traits that enable access to a wider range of resources. We tested this hypothesis with three species of closely related neotropical short-tailed fruit bats (*Carollia*) with overlapping geographic ranges, but with differing degrees of dietary specialization on *Piper* fruits. We applied a new approach, ecological diversity indices, to examine how the *OR*s of these bats vary with increasing ecological specialization.

Measuring diversity among orthogroups provides deeper evolutionary insight than simply comparing numbers of genes and may illuminate the evolutionary processes and functions underlying current diversity in closely related, ecologically similar species. For example, *C. perspicillata* technically has more *OR*s in subfamily *OR*5/8/9 (Figure 1B), but measures of diversity are quite similar across the three species (Figure 2B). In contrast, subfamily *OR*1/3/7 shows substantial differences in diversity among the three species (Figure 2B) even though *C. sowelli* and *C. perspicillata* have quite similar receptor counts (Figure 1B). *OR*s are among the fastest evolving genes in the genome (Yohe *et al.* 2020b), and their turnover via birth-death evolution makes it challenging to compare orthologs among species. For example, there have been so many *OR* gains and losses within rodents that there is less than 70% homology in *OR*s and less than 20% homology in *vomeronasal type-1* genes (another chemoreceptor gene family) between mouse and rat (Zhang *et al.* 2007). The number of receptors only becomes meaningful in terms of describing the “diversity” of receptors in the repertoire, and increased numbers of orthogroups may indicate more potential ligands to be perceived. Thus, if a species has more orthogroups, there are more distinct forms of *OR*s present, and additional paralogs within these orthogroups reinforce the diversity. However, fewer orthogroups and increased paralogs suggest redundancy within an orthogroup. This increased redundancy may suggest selection for retention of similar paralogs, and it potentially has a favorable dosage effect (Teufel *et al.* 2016; Yohe *et al.* 2019b). Tandem gene duplicates are often expressed even greater than twofold, with dramatically higher activity than other sites in the genome (Loehlin and Carroll 2016). Even if increased dosage of expression is not observed, selection for duplicate retention and increased redundancy may also be advantageous if the receptor is critical to detecting a food resource. Olfactory sensory neurons stochastically express a single *OR* gene (Rodriguez 2013; Monahan and Lomvardas 2015), and multiple tandem copies of a gene of similar function may increase the probability of expression. In other words, having multiple copies of a similar receptor may increase its chances of expression. Alternatively, more paralogs may indicate divergent function. While counterintuitive, functional evidence in primates suggests that orthologous *OR*s across divergent species are more likely to bind to the same odorant ligand than paralogs (Adipietro *et al.* 2012). Given the low levels of codon substitution observed in our gene trees, however, we predict that paralogs might be more similar in function and thus we advocate for the dosage effect hypothesis in *Carollia*.

We found that the more generalist frugivorous bat species, *C. perspicillata*, has a more diverse collection of distinct *OR*s compared to the specialist *C. castanea*. To interpret, we assume an increased number of different orthogroups (not number of intact genes) reflects an increased potential to detect different odorant ligands. For example, during the transition from a specialist to a generalist diet in nymphalid butterflies (*Vanessa*), the generalist species expanded their gustatory receptor repertoire and this increased repertoire size is associated with a more diverse plant resource use (Suzuki *et al.* 2018). However, instead of measuring increased gene birth rates, we measure the result of gene duplicate retention as a function of diversity of different receptors in the genome. While the former assumes that duplication rates are deterministic and not stochastic processes, the latter focuses on diversity within orthogroups and may more correctly reflect products of selection. In *Carollia*, because more than 50% of the diet of *C. perspicillata* relies on a diversity of plant resources outside of the genus *Piper* (Figure 1A; *e.g.*, Fleming 1991; Maynard *et al.* 2019), the number of different compounds this species needs to detect may be greater than that of the *Carollia* species that primarily consume fruits within the *Piper* genus. Given the overlapping geographic distributions and dietary niches, divergent olfactory profiles among these *Carollia* species may optimize for the detection of different plant resources in a cluttered rainforest community. We propose this mechanism as a hypothesis that requires further investigation; without a deeper understanding of the plant volatile bouquets of both *Piper* and other plant species, there is certainly the possibility that the fruit volatiles that *Carollia* detects within the *Piper* genus are just as diverse as those across other plant families included in the diet of the generalist.

While which odorant ligands bind to which *OR*s in bats is completely unknown, our analyses constitute a major contribution to help isolate clusters of receptors as candidates for future studies to functionally investigate whether relevant environmental scent cues initiate a response for these receptors. Because total numbers of intact receptors may be irrelevant to olfactory function, exceptional retention of recent gene duplicates and orthogroups containing overrepresentation of species-specific in-paralogs may be a more meaningful starting point for deciphering the ligands for which respective receptors bind. With this approach, instead of attempting to decode hundreds of receptors, our study has narrowed this down to 10–20 genes as good experimental candidates. For example, the *Piper* specialist *C. castanea* shows behavioral preference and attraction to volatile cues of ripened *P. sancti-felicis* fruits (Maynard *et al.* 2019; Leiser-Miller *et al.* 2020). 2-heptanol, for example, shows a strong signature of both *C. castanea* detection and abundance in *Piper* highly consumed by these bats (Leiser-Miller *et al.* 2020; Santana *et al.* 2021). Thus, a future study may test the hypothesis that receptors demonstrating exceptional redundancy within *C. castanea* [*e.g.*, such as those found in *OR*4 (Figure 2B) or *OR*10 (Figure 3)] respond to volatiles of ripened fruits such as 2-heptanol of *P. sancti-felicis* in a biochemical assay.

Detecting olfactory adaptation at the molecular level in olfaction remains an open challenge (Yohe and Brand 2018). Interpretation of our results includes several underlying assumptions. For example, because *OR* data were generated using targeted bait capture, highly divergent *OR*s that were not expressed may not have been sequenced. However, our approach obtained about five times more *OR* genes for *Carollia* than previous studies (Hayden *et al.* 2014). Past 20% sampling effort estimated that *Carollia perspicillata* would have 954 expected receptors (Hayden *et al.* 2014), of which the authors had only sequenced 194. We recovered 1115 intact receptor genes for this species, which is a reasonably comparable number to the expected given our completely *de novo* approach. Another caveat includes the difficulty in deciphering in-paralogs from allelic diversity, of which the latter is likely vastly underestimated (Yoder and Larsen 2014). Finally, we interpret redundancy within an orthogroup as more dosage, but it is entirely possible that a single amino acid change within a duplicate pair of receptors may result in different ligand interaction and potentially divergent behavioral responses with a given odorant. Distinguishing the two in large gene families continues to be a confounding issue requiring exceptionally high coverage to characterize read mapping bias of duplicates and high-quality reference genomes to map flanking regions of duplicate regions, both outside the scope of this analysis. With these assumptions in mind, our discovery of inverse patterns of dietary specialization and *OR* diversity may have consequential implications for understanding how evolution shapes complex and rapidly evolving gene families.

## Data availability

Raw Illumina sequence reads from targeted sequence capture were deposited to GenBank Sequence Read Archive under BioProject PRJNA531931, BioSamples SRX11499917-19, and sequence accessions SRR15193284-86. Alignments, sequence baits, data sets, and R scripts that reproduce the analyses and figures were deposited into figshare: https://doi.org/10.25387/g3.14665179.
